# Reasons and Features of Patients Who Leave the Emergency Department Without Being Seen

**DOI:** 10.1155/emmi/7199212

**Published:** 2025-01-11

**Authors:** Fahad Abuguyan, Abdulaziz Alhusainy, Omar Alsuliman, Sarah Alqahtani, Abdulrahman Alrajhi

**Affiliations:** ^1^Department of Emergency Medicine, College of Medicine, King Saud University, Riyadh, Saudi Arabia; ^2^Emergency Medicine Department, National Guard Health Affairs, King Abdulaziz Medical City, Riyadh, Saudi Arabia

**Keywords:** emergency, emergency department, left without being seen, patients, triage

## Abstract

**Background:** Emergency medicine practitioners encounter significant challenges related to patients who leave emergency departments (EDs) without being seen (LWBS) in the ED. We aimed to assess the characteristics, reasons, and rate of patients who left without being seen in the tertiary teaching hospital ED of King Khalid University Hospital in Riyadh, Saudi Arabia.

**Methods:** A qualitative prospective observational study was conducted from January 4, 2023, to May 17, 2023, among patients who left the ED without being seen in the King Khalid University Hospital, King Saud University Medical City, a tertiary hospital in Riyadh, Saudi Arabia. Data were collected from the ED administrative database, phone surveys, and electronic files of the identified patients. Phone interviews with questionnaires were conducted with patients participating in the study within 1 week of their ED visit.

**Results:** During the study period, 16,682 patients visited the adult ED and 636 (3.81%) remained unseen; 300 patients met the study criteria. Of these, 288 (96%) arrived at the hospital via private car and 12 (4%) used ambulances. Trauma and gastrointestinal, neurological, and cardiovascular complaints were the most common, reported by 24.33%, 18.33%, 12%, and 10% of patients, respectively. In our study, 55 patients (18.3%) experienced prolonged waiting times of more than four hours before leaving the ED. Most patients (75%) inquired about the reasons for not being seen by a physician while waiting; 137 (45.6%) asked a receptionist, 117 (39%) asked a nurse, and 28 (9.3%) asked a doctor. According to 76 (25.3%) patients, they should not have to wait, whereas 82 (27.3%) said that they should wait for an hour. When asked whether they would visit the same ED in the future, 213 (71%) answered yes and 87 (29%) answered no.

**Conclusion:** We conclude that in our center, prolonged waiting time and ED overcrowding are the main reasons why patients leave the ED without seeing a physician. Younger patients are more prone to LWBS, with trauma and gastroenterological complaints being the most common presenting symptoms. The LWBS rate was 3.81% of the total ED visits during the study period.

## 1. Introduction

Patients who leave without being seen (LWBS) in the emergency department (ED) depart after registering in the patient's area but before undergoing assessment by an emergency medicine physician [1]. Emergency medicine practitioners encounter significant challenges related to LWBS in the ED [2]. Overcrowding serves as an indirect marker of ED performance, contributing to an increase in the number of patients leaving without seeing a physician and diminishing overall patient satisfaction. These individuals face an elevated risk of morbidity and mortality because of their underlying medical conditions [3].

While a substantial proportion of patients with LWBS present with low acuity and severity triage scores, some are susceptible to complications, worsening conditions, or, in extreme cases, life-threatening consequences leading to mortality [4–6]. Numerous studies have demonstrated that individuals who leave the ED without seeing physicians are at a heightened risk of revisiting the ED and seeking care at other healthcare facilities shortly after, with some requiring immediate admission [1, 5–11]. An increase in the number of LWBS patients is associated with adverse outcomes, including ED overcrowding, prolonged waiting times, diminished service quality, decreased patient satisfaction, and increased violence against healthcare professionals [4, 12–14]. Consequently, a considerable variation is observed in LWBS rates in the existing literature, ranging from 0% to 20.3% worldwide and from 0.8% to 9.8% in the Saudi Arabian population [1, 4–6, 8–10, 15–20].

A key quality indicator for the EDs is the percentage of patients with LWBS relative to the total number of ED visits [19]. This indicator varies among institutions and is influenced by factors such as ED flow, total number of ED visits, and institutional type. Studies consistently show an increase in LWBS rates in crowded EDs and specific types of institutions, including teaching hospitals such as our own [4, 12, 14]. Multiple investigations have identified prolonged waiting times as the primary reason for LWBS, with additional factors encompassing patients' perceived improvement, work and home commitments, dissatisfaction with hospital staff, lack of patient-team communication, and financial and insurance coverage issues [1, 5–7, 9, 10, 21].

The limited data reporting the issue of patients with LWBS have constrained current efforts to investigate this phenomenon. The existing literature predominantly focuses on patient-level or operational determinants at individual hospitals [22, 23]. The scarcity of information hampers decision-makers' ability to comprehend how crowding affects vulnerable groups and develop system-level measures to enhance emergency treatment access. Given the significance and impact of LWBS patients on healthcare facilities and ED quality of care, we aimed to explore the reasons, characteristics, and rate of LWBS patients in a tertiary academic hospital at King Khalid University Hospital in Riyadh, Saudi Arabia.

## 2. Methodology

This qualitative prospective observational study was conducted from January 4, 2023, to May 17, 2023, among patients who left without seeing a physician in the ED of King Khalid University Hospital, King Saud University Medical City, a tertiary hospital in Riyadh, Saudi Arabia. The ED nurse screener assessed each patient arriving with a caretaker or independently, triaged them to the appropriate Canadian Triage and Acuity Scale (CTAS) category, recorded their vital signs, and directed them to the waiting area. Upon the patient's turn, the nurse called them, and if unanswered after three attempts, the patient was labeled as “left without being seen.” Patient records were regularly monitored by the EDs.

The inclusion criteria included all adult patients aged > 14 years who left the ED without being examined by a physician during the study period. The exclusion criteria were pediatric patients below 14 years of age, patients exceeding 20 weeks of pregnancy, and those with pure obstetric or gynecological complaints seen in the obstetrics–gynecology ED.

Of the 636 patients, 336 were excluded for various reasons, leaving 300 for inclusion. Data were collected from the ED administrative database, phone surveys, and electronic patient files. Phone interviews with willing participants were conducted within 1 week of the ED visit. Informed consent was obtained from all participants. The questionnaire was adapted from Fernandes et al.'s study titled “Emergency department patients who leave without seeing a physician: The Toronto Hospital experience” [10].

In addition to the questionnaire variables, supplementary factors including age, sex, CTAS level, day of visit, complaint category, shift period (morning, evening, or night), and mode of arrival (private car or ambulance) were incorporated. IBM SPSS 28 for Windows was used for the statistical analysis. We employed the chi-square test, Student's *t*-test, one-way analysis of variance, and Pearson's correlation tests, as appropriate for the study and outcome variables. Statistical significance was set at *p* value < 0.05 to ensure the precision of the results. Descriptive statistics, including means, standard deviations, frequencies, and percentages, were used to present categorical variables.

## 3. Results

**Figure 1:**
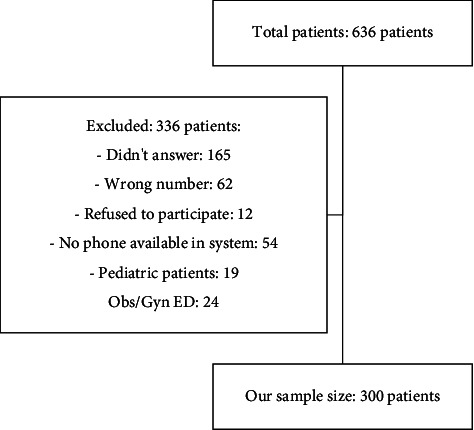
Sample size evaluation.

During the study period, 16,682 patients sought care in the adult ED of King Khalid University Hospital in Riyadh, Saudi Arabia. Of these, 636 patients (3.81%) were left unseen and 300 patients who met the inclusion criteria were included in this study (Figure 1). The sex distribution among the patients who were left unseen was equal, with each sex accounting for 50% of the total LWBS patients. The median age of the patients in our study was 34 years, ranging from 14 to 90 years. Private cars were the predominant mode of transportation used by 288 patients (96%), whereas 12 patients (4%) used ambulances (Table 1). The analysis of our LWBS patient population revealed that the highest number of visits occurred on Mondays, with 58 patients (19.3%), followed by Thursdays (51 patients, 17%) and Fridays (weekends), which had the lowest number of visits (4.3%). The remaining patients visited the hospital on alternate weekdays. Regarding shift periods, 148 patients (49.3%) visited the hospital during the evening shift (3 PM–11 PM), followed by the night shift (11 PM–7 AM) and 90 patients (30%) visited the morning shift (7 AM–3 PM) with 62 patients (20.7%) (Table 1). The analysis of the patients' CTAS categories revealed that 236 patients (78.7%) were in CTAS Category 3, indicating a nonimmediate life-threatening condition requiring attention within 2 h. This was followed by 60 patients (20%) in CTAS Category 4, three patients (1%) in CTAS Category 5, and one patient (0.3%) in CTAS Category 2.

Trauma and gastrointestinal, neurological, and cardiovascular complaints were most common, reported by 24.33%, 18.33%, 12%, and 10% of patients, respectively (Table 2). The remaining patients presented with complaints related to cardiovascular, musculoskeletal, infectious, urology–nephrology, pulmonary, endocrine, oncology, gynecology, hematology, psychological, ENT, autoimmune, dermal, and vascular conditions. A significant proportion of patients (55.3%) reported having problems for less than 24 h before arriving at the hospital, with 70 patients (23.3%) and 64 patients (21.4%) reporting problems persisting for more than 72 h and 24–72 h, respectively. Regarding the distance from their houses to the hospital, most patients (37.3%) reported that the hospital was 10–20 min away, followed by 21.7%, 18.7%, 13%, and 9.3% reporting distances of 20–30 min, less than 10 min, > 40 min, and 30–40 min, respectively (Table 3).

A considerable proportion of patients (74%) had visited the hospital previously, while 26% had visited the hospital for the first time. In addition, 53.7% did not visit the hospital by themselves and 46.3% arrived independently. A notable proportion of patients (18.3%) waited for more than 4 hours before leaving the ED. While waiting, 75% of the patients inquired about the reason for not being seen by the physician yet, with the majority (45.6%) asking a receptionist, 39% asking a nurse, and 9.3% asking a doctor (Table 3). The results showed that 72.7% of patients who inquired about their wait times received an explanation. Regarding the factors influencing their decision to leave the ED, 80% of the patients reported leaving because of fatigue from waiting, while 24.3% believed their problem could wait. A significant proportion of patients (50.3%) perceived the ED as very busy, and 26.3% expressed dissatisfaction with the hospital staff (Table 3).

Following their departure from the ED, 61.7% of the patients sought medical attention elsewhere, with the majority seeking care within 24 h. Of those seeking further medical attention, 61.0% went to another ED, 16.0% revisited our ED, 13.0% went to a primary care clinic, and 10% sought care at a specialized clinic.

When questioned about waiting times, 25.3% of the patients believed that they should not have to wait at all, while 27.3% believed that waiting for an hour was acceptable. Regarding future visits, 71% of patients expressed their intention to revisit the same ED, whereas 29% indicated otherwise.

## 4. Discussion

Patients who leave the ED without seeing a doctor (LWBS) may face heightened risk of complications, worsening health conditions, and potentially fatal outcomes [4]. We aimed to scrutinize the characteristics and reasons for LWBS in patients at King Khalid University Hospital (KKUH) in Riyadh, Saudi Arabia. The analysis revealed that most patients arrived at the hospital in private car, indicating stable medical conditions suitable for private transportation. Patients with more severe conditions may have opted for ambulance transport because the research suggests that ambulance arrivals are less likely to result in departures [24].

Our study period showed an LWBS rate of 3.81% of the total ED visits, aligning with global and local studies reporting LWBS rates ranging between 0% and 20.3% and 0.8% and 9.8%, respectively [1, 4–6, 9–11, 15–20, 24]. After scrutinizing the CTAS data, we found that 78.7% of the patients who were left unseen had urgent medical conditions categorized under CTAS Category 3, requiring prompt attention. This contrasts with the results of another local study reporting only 11.9% of CTAS Category 3 cases among LWBS patients [16] and a Canadian study reporting 26.6% [9]. In our study, we found that only one patient was categorized under CTAS Category 2, and upon reviewing the medical records, we found that the patient was in the waiting area because our radius was occupied by a greater number of sick patients at that time. The accuracy of the triage system is debatable, with patients in our hospital sharing the same category as those in previous studies, suggesting either access to alternative medical care institutions or leaving because of long waiting times at KKUH. Lambe et al. estimated an average waiting time of 56 min before being seen, with 42% waiting longer than an hour, attributing extended waits to low proportions of ED doctors and triage nurses [25].

Significantly, 18.3% of patients in our study experienced waiting times exceeding 4 h before leaving the ED, potentially contributing to their decision to leave without seeing a physician. Comparable findings were reported in a previous study in conducted in teaching hospitals, linking longer treatment and waiting times to trainee education and a high risk of LWBS [1]. These results underscore the profound impact of waiting time on patient satisfaction with emergency care services, a global issue in developing countries that leads to frustration and dissatisfaction, and compels patients to leave before receiving care [21, 26].

The causes of LWBS are multifaceted, encompassing population expansion, a decline in the number of primary healthcare professionals, prolonged wait times for community surgeries and medical imaging, and the availability of ED services 24/7 [27]. Despite the lack of consensus in the literature on the association between less urgent patient categories and ED congestion, addressing patient perceptions of workload and reducing overcrowding can enhance patient satisfaction and ensure necessary care [28].

Mistriaging is often observed in the emergency field, especially in institutions where the ED triaging is handled by nonphysician health practitioners [18]. However, overtriaging is generally better in many cases because it significantly decreases costs [20].

In addition, 23.3% of patients reported having complaints for more than 72 h before coming to KKUH, suggesting stability and the ability to wait longer before seeking medical care. Among those leaving, 80% reported leaving because of fatigue from waiting, whereas 24.3% felt that their condition caused them to wait longer at home. Regarding the time of patient visits, more patients left the ED without being seen during the evening shift, possibly because of increased patient crowds and the convenience of nonurgent visits during this time. Consequently, the findings of this study align with the medical managers' view that the triage regimen may need to be reconsidered to accommodate the changing situation and population needs. For triage standardization in the ED, the Emergency Severity Index (ESI), Manchester Triage System (MTS), and South African Triage Scale (SATS) can be considered in applicable programs for improving healthcare performance. However, the main determinant was adaptability to the present situation; the present situation in the ED of a tertiary hospital was characterized in this study.

LWBS is associated with presentation time, with previous studies reporting higher rates in summer or fall and during the evening or overnight shifts [4, 29, 30]. Our study revealed lower LWBS rates on weekends (Fridays and Saturdays), contrary to the findings of Hobbs et al., who observed higher rates over the weekends [31].

The perception of ED being very busy influenced 50.3% of patients to leave without seeing a physician, underscoring the need to address patient perceptions, alleviate overcrowding, and reduce long wait times. Following departure, 77% sought medical care in the ED, with most opting for a second visit to our department or another hospital. Of those seeking care elsewhere, 23% sought help in a clinic, highlighting potential barriers such as financial constraints and clinic availability.

The study revealed diverse patient expectations, with 25.3% believing that they should not have to wait at all and 27.3% expecting to wait for an hour. Unexpectedly, 71% of the patients who left the ED without being seen expressed an intention to return if they needed medical care in the future, suggesting a retained level of trust in the hospital's capacity to provide care. The availability of full eligibility and insurance, along with the choice of another medical institution, may have influenced their decisions.

### 4.1. Limitations

The inability to evaluate the results of patients who underwent LWBS constitutes the primary research limitation. We recognize that this kind of evaluation would provide information on the patients' progress toward their morbid conditions, and any mortality that may occur. Lack of resources made it difficult to include this follow-up evaluation in the present investigation. We hope that future research will consider this aspect.

Second, the study was limited by the number of patients involved in the study, and the accessibility of the patients was somewhat difficult due to unanswered phone call interviews. We interviewed the patients using phone within 1 week of the ED visit to limit the possibility of forgetting the ED visit details. The actual causes of LWBS could not be determined; however, the questionnaire employed in this study attempted to identify the elements leading to it.

In addition, the data were limited to one medical center, in which the factors behind LWBS in the current patient population are not necessarily applicable to other institutions. Further multicenter studies with larger sample sizes are required to overcome these limitations. Evaluation of the Emergency Medicine Department in association with the outpatient general practice in our hospital to reduce waiting time is recommended in future studies. Furthermore, the triage system in the current study was performed by nonphysician staff, which might have overrated the patients' CTAS category.

## 5. Conclusion

We concluded that in our center, the main reasons for patients leaving the ED without being seen by a physician were prolonged waiting times and ED overcrowding. We found that younger patients were more prone to developing LWBS. Trauma and gastroenterological complaints were the most common complaints in our study population. The proportion of patients with LWBS was 3.81% of the total visits to the ED during the study period. This raises the question of efforts toward ameliorating the quality of emergency care and possible ways to improve it. This study provides valuable information regarding the CTAS and complaint categories of patients who visited the hospital. It could be useful for healthcare providers in prioritizing patients based on the severity of their condition, and for policymakers in planning and managing healthcare services.

## Figures and Tables

**Table 1:** Characteristics of patients (*N* = 300).

	*N*	%
*Sex*		
Female	150	50
Male	150	50

*Age (in years)*		
14–20	40	13.3
21–30	84	28
31–40	67	22.3
41–50	32	10.7
51–60	37	12.3
61–70	26	8.7
> 71	14	4.7

*Mode of arrival*		
Ambulance	12	4
Walk-in (private car)	288	96

*Shift period*		
Morning (7 AM–3 PM)	62	20.7
Evening (3 PM–11 PM)	148	49.3
Night (11 PM–7 AM)	90	30

*Days of visits*		
Sunday	46	15.3
Monday	58	19.3
Tuesday	37	12.3
Wednesday	48	16
Thursday	51	17
Friday (weekend)	13	4.3
Saturday (weekend)	47	15.7

**Table 2:** Canadian triage and acuity scale.

	*N*	%
*CTAS category*		
2	1	0.3
3	236	78.7
4	60	20
5	3	1

*Complaint category*		
Trauma	73	24.33
Gastrointestinal	55	18.33
Neurological	36	12.00
Cardiovascular	30	10.00
Musculoskeletal	23	7.67
Infectious	22	7.33
Urology–nephrology	18	6.00
Pulmonary	9	3.00
Endocrine	6	2.00
Oncology	5	1.67
Gynecology	5	1.67
Hematology	4	1.33
Psych	3	1.00
Ear, nose, and throat	3	1.00
Autoimmune	3	1.00
Dermatological	3	1.00
Vascular	2	0.67

**Table 3:** The Toronto hospital questionnaire.

	*N*	%
*How long did you have the problem before you came to the hospital?*		
Less than 24 h	166	55.3
24–72 h	64	21.3
More than 72 h	70	23.3

*Approximately how far is the hospital from your house?*		
Less than 10 min	56	18.7
10–20 min	112	37.3
20–30 min	65	21.7
30–40 min	28	9.3
More than 40 min	39	13.0

*Did you come to the hospital by yourself?*		
No	161	53.7
Yes	139	46.3

*Was this your first visit to the hospital?*		
No	222	74.0
Yes	78	26.0

*After registering, how long did you wait before leaving the ED?*		
0 h	5	1.7
15 min	12	4.0
30 min	27	9.0
1 h	64	21.3
2 h	55	18.3
3 h	46	15.3
4 h	36	12.0
More than 4 h	55	18.3

*While you are waiting to be seen by a doctor, did you ask anyone how long it would be before you were seen?*		
No	75	25.0
Yes	225	75.0

*Whom did you ask?*		
A doctor	28	9.33
A nurse	117	39
A receptionist	137	45.67
Another patient	18	6

*Did any of the people you spoke to explain to you why you had not been seen yet?*		
No	82	27.3
Yes	218	72.7

*Factors that influenced your decision to leave the ED*		
*I was tired of waiting*		
Don't know	8	2.6
No	52	17.3
Yes	240	80.0

*I felt my problem could wait*		
Don't know	12	4.0
No	215	71.7
Yes	73	24.3

*The ED appeared to be very busy*		
Don't know	15	5.0
No	134	44.7
Yes	151	50.3

*I was unhappy with the hospital staff*		
Don't know	41	13.6
No	180	60.0
Yes	79	26.3

*I was unhappy with the other patients*		
Don't know	43	14.3
No	227	75.7
Yes	30	10.0

*I had to go back to home/work/school*		
Don't know	19	6.4
No	210	70.0
Yes	71	23.7

*After leaving, did you seek medical attention elsewhere?*		
No	115	38.3
Yes	185	61.7

*Within which time period did you seek this additional medical attention (from leaving our ED)?*		
Less than 24 h	253	84.33
24–72 h	39	13.0
More than 72 h	8	2.67

*Where did you seek further medical attention?*		
Another ED	183	61.0
Our ED	48	16.0
Primary care clinic/center	39	13.0
Specialized clinic	30	10.0

*How long do you think that someone with your problem should have to wait in an ED before seen by a doctor?*		
0 h	76	25.3
15 min	36	12.0
30 min	63	21.0
1 h	82	27.3
2 h	29	9.7
3 h	12	4.0
4 h	2	0.7

*Would you return to this ED in the future?*		
No	87	29.0
Yes	213	71.0

## Data Availability

The data of this study are derived from patient phone calls and hospital electronic healthcare system. Due to privacy and confidentiality agreements, the raw data are not publicly available but are available from the corresponding author upon reasonable request and with appropriate ethical approval.
